# Zero-Sum Construal of Workplace Success Promotes Initial Work Role Behavior by Activating Prevention Focus: Evidence From Chinese College and University Graduates

**DOI:** 10.3389/fpsyg.2020.01191

**Published:** 2020-06-30

**Authors:** Haiyan Zhang, Shuwei Sun

**Affiliations:** ^1^Business School, Jiangsu Normal University, Xuzhou, China; ^2^School of Mathematics and Statistics, Xuzhou University of Technology, Xuzhou, China

**Keywords:** zero-sum construal of workplace success, initial work role behavior, prevention focus, pay level, Chinese college and university graduates

## Abstract

Given that the population of Chinese college and university graduates has become larger and larger year by year since 1999, the problem of individual graduate’s employment and workplace adaptability has captured widespread social concern and interest from both scholars and practitioners. Initial work role behavior is essential to an individual graduate’s successful adaptation to the workplace and sustainable career development as a new employee. Based on social cognition theory, sense-making theory, and regulatory focus theory, we argue that the individual graduate’s zero-sum construal of workplace success is a key factor influencing his or her initial workplace adaptability, which not only directly augments initial work role behavior but also elicits the new employee’s inclination toward prevention focus, which in turn enhances initial work role behavior in the context of China’s present steady economic development. Moreover, the individual graduate’s average pay level moderates the mediating effect of prevention focus, such that the higher the average pay level is, the stronger the positive effect of zero-sum construal on initial work role behavior via prevention focus becomes. Two-stage survey data from 258 Chinese university graduates who have already entered organizations as full-time employees and their direct supervisors provided evidence consistent with our hypothesized first-stage moderated mediation model. Implications, limitations, and future research suggestions are also discussed.

## Introduction

Since the expansion of enrollment into Chinese colleges and universities in 1999, the problem of individual graduate’s adaptability to employment and the workplace has always been a hot topic and has drawn widespread attention around China. On January 16, 2019, the Ministry of Human Resources and Social Security of the People’s Republic of China stated that the number of college and university graduates would reach 8.34 million in 2019, which goes beyond the 8.2 million of 2018 and creates a new record. Meanwhile, an investigation from LinkedIn indicated a sharp decline trend among different generations in their average on-job time in their first job, such that employees born in the periods 1970–1979, 1980–1989, 1990–1994, and 1995–present, respectively persisted in their first job for 51, 43, 19, and 7 months, on average. On the one hand, a huge number of graduates are flocking into human resource market continuously, which in turn leads to unprecedentedly fierce job-seeking competition in China; on the other hand, many new employees hastily leave organizations and re-enter the competitive job-hunting market, mainly because of a lack of adaptability in their initial work role behavior. Both of these factors exacerbate the severity of the employment environment for Chinese college and university graduates, hamper their organizational socialization, and threaten their sustainable career development. Consequently, how to unravel the adaptability mechanism of individual graduate’s initial work role behavior as a new employee and how to put forward effective strategies to enhance his or her initial work role behavior have become increasingly severe, urgent, and significant problems in the eyes of both scholars and practitioners in China.

There is no doubt that numerous factors affect individual graduate’s initial job choices, work role behavior decisions, and turnover intention, such as social values and employment culture (Agarwala, 2008), unemployment ratio and job prospects (Athanasou, 2003), pay level and pay dispersion (He et al., 2016), leader-member relationship (Bauer et al., 2006), growth and career opportunities (Azizzadeh et al., 2003), work environment (Katherine et al., 2012), family time (Katherine et al., 2012), or the skills, competencies, and abilities of the individual (Agarwala, 2008). However, based on social cognition theory (Niedenthal et al., 1985; Baldwin, 1992; Fiske, 1992) and sense-making theory (Weick, 1995), what effect the above factors (and other potential factors not mentioned here) will exert on individual employee’s daily workplace behavior and turnover decisions largely depends on his or her general cognitive construal of them. We are very interested in individual graduate’s initial adaptability to work as a new employee in the context of China’s present steady economic development. Hence, our research seeks to unravel the adaptability mechanism from the perspective of zero-sum construal of workplace success and prevention focus.

“Zero-sum construal of workplace success” is a fundamental cognitive interpretation of workplace success that refers to an individual employee’s general sense-making of whether employee A’s success signifies Bs’ failure or loss in the workplace (Foster, 1965; Esses et al., 1998). Sirola and Pitesa (2017) found that when an individual employee was deciding whether to engage in helping behavior in the workplace, his or her zero-sum construal of success arising from economic downturns would generate a heavy negative influence, such that the stronger individual employee’s zero-sum construal of success was, the less likelihood there was that he or she would help a coworker(s), whereas, when an individual employee was inclined to construe success as a win–win or a positive-sum game, he or she tended to provide help in the workplace. Compared to Sirola and Pitesa (2017)’s research, our research has two important distinctions. First, we aim at unraveling the adaptability mechanism of an individual graduate’s initial work role behavior as a new employee in the context of China’s present unprecedentedly fierce job-seeking competition during a steady economic development period rather than a difficult economic period. When an economic downturn takes place, the worsening of the economy causes the chances of either getting jobs or raising wealth to fall sharply on average, which in turn may make an individual’s zero-sum construal of success salient and reduce his or her likelihood to help others. In the context of China’s present steady economic development, however, a large number of vacant posts need to be filled urgently. In this situation, the large population of job seekers in China is the main reason for the fierce job-seeking competition. Therefore, only candidates with strong abilities, knowledge, and skills can win and obtain jobs or other workplace success. In brief, in China’s present context with plenty of job opportunities, an individual’s zero-sum construal of success, which substantially arises from the fierce job-seeking competition due to the huge numbers of graduates, is very different from that mainly coming from the much lower likelihood of getting jobs or raising wealth during a difficult economic period. Second, we focus on initial work role behavior rather than workplace helping behavior. Initial work role behavior emphasizes the new employee’s adaptability to the workplace, which reflects the extent to which the new employee copes with, responds to, and supports changes that influence his or her work roles, which are usually specified by the job description or supervisors’ expectations. In essence, it is often attributed to in-role behavior, which, to some extent, could be regarded as a kind of egoistic behavior. Workplace helping behavior, however, is essentially a kind of organizational citizenship behavior focusing on voluntarily assisting coworkers (Sparrowe and Kraimer, 2006), which is often considered as typical altruistic behavior. Accordingly, the outcome variable we are interested in is very different from that in Sirola and Pitesa (2017)’s research.

We wonder, compared to the negative influences confirmed by Sirola and Pitesa (2017), whether the above two important distinctions could cause individual graduate’s zero-sum construal of workplace success to demonstrate different influences on his or her initial workplace adaptability in China’s context, why, and under what boundary condition. On the basis of social cognition theory (Niedenthal et al., 1985; Baldwin, 1992; Fiske, 1992) and sense-making theory (Weick, 1995), we speculate that individual graduate’s zero-sum construal of workplace success in China’s context may have “positive” effects. Unfortunately, most of the extant literature tends to emphasize the “negative” roles of zero-sum construal (Foster, 1965; Esses et al., 1998; Norton and Sommers, 2011; Sirola and Pitesa, 2017), with little attention and few efforts dedicated to examining the potential “positive” roles, which is not conducive to our comprehensive understanding of zero-sum construal. Accordingly, our study aims to explore “what effect” individual graduate’s zero-sum construal of workplace success actually exerts on his or her initial work role behavior as a new employee, “why,” and “under what boundary condition.”

We seek to unravel the black box of the mechanism of individual graduate’s initial workplace adaptability in view of prevention focus based on regulatory focus theory (Higgins, 1997; Higgins, 1998). Prevention focus, one of the two basic self-regulation systems and motives of human beings, primarily concentrates on prevention goals and punishment-avoidance (Kark and Van Dijk, 2007). It is found that situational factors are the main triggers of an individual’s inclination toward prevention focus (Higgins, 1997, 1998). Though zero-sum construal of workplace success is not a kind of situation, it is exactly the individual graduate’s general cognition of situations where workplace success events take place. Once shaped, the individual’s zero-sum construal of workplace success will efficiently guide his or her workplace behavior in a less time- and energy-consuming way (Niedenthal et al., 1985; Baldwin, 1992; Fiske, 1992; Weick, 1995), as it is impossible and generally non-economic to exhaustively scrutinize every situation where workplace success occurs (Macrae et al., 1994). Further, a zero-sum construal of workplace success emphasizes that employee A’s workplace success is the workplace loss or failure of other employees, which is exactly consistent with prevention focus’s main concern with prevention goals and punishment-avoidance. Thus, individual graduate’s zero-sum construal of workplace success may be one triggering factor that elicits his or her prevention focus inclination. After being activated by the individual graduate’s zero-sum construal of workplace success, prevention focus is more likely to motivate the new employee to concentrate on what he or she “should” do in the initial workplace to avoid potential loss or failure, since this is the main concern and goal of an individual employee who operates primarily within a stronger prevention focus inclination. The individual graduate’s initial work role behavior as a new employee, which could reflect his or her workplace adaptability, is just what he or she “should” do in the workplace. Thereby, the individual graduate’s prevention focus evoked by his or her zero-sum construal of workplace success may promote his or her initial work role behavior as a new employee. The findings of the extant literature that an individual’s prevention focus is positively related to his or her in-role performance (Neubert et al., 2008) could provide direct evidence for this inference. Taking the above two inferences together, we identify prevention focus as one of the potential mediators in the relationship between an individual graduate’s zero-sum construal of workplace success and his or her initial work role behavior. Moreover, as the main part of the individual graduate’s opportunity cost of failure in a workplace zero-sum game, we speculate that his or her average pay level may influence prevention focus’s mediation in the association between zero-sum construal of workplace success and initial work role behavior. Therefore, our study explores its possible moderation role in prevention focus’s mediating process as well.

After putting forward the research problem in section “Introduction,” section “Theoretical Background and Hypotheses” reviews the theoretical background and constructs a first-stage moderated mediation model. Section “Materials and Methods” depicts the research sample, data collection, measurements, and data analysis methods. Section “Results” presents the empirical findings and hypothesis testing results. Theoretical and practical implications, as well as limitations and suggestions for future research, are discussed in section “Discussion.”

## Theoretical Background and Hypotheses

### Zero-Sum Construal of Workplace Success and Its Effect on Initial Work Role Behavior

According to Foster (1965) and Esses et al. (1998), “zero-sum construal of success” is defined as an individual’s general cognition, interpretation, or making-sense of whether person A’s success represents person Bs’ loss or reduced probability of succeeding. Success here refers to a wide variety of things, such as attaining wealth, making job achievements, social status improvement, or even winning a match or contest. Based on social cognition theory (Niedenthal et al., 1985; Baldwin, 1992; Fiske, 1992) and sense-making theory (Weick, 1995), we can speculate that zero-sum construal of success has two main features. First, it shows remarkable differences between individuals, such that people with different backgrounds and experiences could have distinct levels of zero-sum construal of success. For example, some people may consider social wealth just as a zero-sum system, whereas other people may view it as a non-zero-sum system that could be enlarged through technological innovation (Sirola and Pitesa, 2017). Second, as one of the individual’s fundamental ways of making sense of the world, once formed, zero-sum construal of success will play a significant role in the individual’s daily behavior and decision-making (Weick, 1995), since it is impossible and generally non-economic to exhaustively scrutinize every situational detail (Macrae et al., 1994). For instance, Esses et al. (1998) confirmed that zero-sum belief was a vital predictor of one’s perception of competitiveness within a team, and Sirola and Pitesa (2017) found that economic downturns could lead to an individual’s salient zero-sum belief of success, which thereby undermined his or her workplace helping behavior.

As for our study, we focus on Chinese college and university graduates, who are the majority of Chinese organizations’ new employees, and we are particularly interested in the individual graduate’s zero-sum construal of “workplace success,” which is gradually shaped once he or she graduates from college or university and enters an organization as a new employee. We suppose, based on social cognition theory (Niedenthal et al., 1985; Baldwin, 1992; Fiske, 1992) and sense-making theory (Weick, 1995), that the individual graduate’s zero-sum construal of workplace success may be an important factor influencing his or her initial workplace adaptability. Thus, we choose “zero-sum construal of workplace success” as the independent variable and “initial work role behavior” as the dependent variable, and then infer the potential relationship between them.

By the definition of “zero-sum construal of success,” individual graduate’s “zero-sum construal of workplace success” refers to his or her general cognition or interpretation of whether graduate/employee A’s workplace success means graduate/employee Bs’ workplace loss or lower likelihood of obtaining workplace success. As a new employee, “workplace success” usually comprises getting the desired job, successfully passing the probationary period, obtaining a job promotion, getting a pay-raise, and the like. Despite that there are plenty of job opportunities due to China’s present steady economic development, a fact that cannot be ignored is that China has an extremely large job demanding population. As the population of Chinese college and university graduates has become larger and larger year by year since the enrollment expansion in 1999, the competition in the job-hunting market has become more and more fierce. According to social cognition theory (Niedenthal et al., 1985; Baldwin, 1992; Fiske, 1992) and sense-making theory (Weick, 1995), however, only through individual graduate’s cognition or making-sense of workplace success under the situations of the competitive job-hunting market and the new workplace, which is full of ambiguity, uncertainty, and complexity, could the situations play roles in the individual graduate’s initial work role behavior. Moreover, the individual’s cognitive construal based on sense-making is an effective way to cope with the ambiguity, uncertainty, and complexity around him or her, which in turn could facilitate his or her workplace adaptability (Macrae et al., 1994). Therefore, for the sake of smoothly interacting with the uncertain external working situations and quickly adapting to the new workplace, the individual graduate will unconsciously make sense of the linkage between one’s behavior and outcome in the job-hunting market or in the workplace, which thereby gradually shapes his or her construal of workplace success/failure. The construal of success/failure unconsciously shaped during the sense-making process of success in the job-hunting market or in the workplace based on his or her own experience and interactions with peers (Louis, 1980) will then automatically serve to guide his or her subsequent work behavior (Weick, 1995). Specifically, based on the views of sense-making theory (Weick, 1995), if the individual graduate tends to construe workplace success in a zero-sum manner and concludes that successful job hunting, passing the probationary period smoothly, obtaining a job promotion, or getting a pay-raise by another usually implies his or her reduced probability of achieving success in these respects, this making-sense or interpretation will undoubtedly enhance his or her competition awareness in the initial workplace and lead to the formation of zero-sum construal of workplace success. In the context of China’s current steady economic development, a large number of vacant posts exist and need to be filled urgently. However, as stated above, the population of job-demanding individuals in China is so large that the competition between job seekers is unprecedentedly fierce. Therefore, only candidates with strong knowledge, abilities, and skills can land jobs or achieve other workplace successes. Thus, we believe that zero-sum construal of workplace success in the context of China’s present steady economic development with abundant job opportunities is very different from that mainly stemming from economic downturns (Sirola and Pitesa, 2017) and is more likely to drive the individual graduate to cherish his or her job, which was hard-won against fierce competition, and to focus on how to gain a foothold at the initial working position and then continuously strengthen his or her competitiveness in the follow-up zero-sum games, such as passing the probationary period, obtaining a job promotion, or getting a pay-raise. As a new employee, initial work role behavior is so crucial to whether a person gains a foothold at the initial working position that the individual graduate must pour himself or herself into it to quickly and effectively adapt to the new job and environment. This is just as the saying goes, “Learn to walk before you learn to run.” Accordingly, we posit that an individual graduate’s zero-sum construal of workplace success is likely to positively influence his or her initial work role behavior as a new employee.

*Hypothesis 1*: Individual graduate’s zero-sum construal of workplace success has a positive effect on his or her initial work role behavior as a new employee.

### Mediation Role of Prevention Focus

As workplace adaptability demands that individual graduates invest a lot of resources, including attention, time, and effort, for the fulfillment of responsibilities and obligations to gain a foothold at the initial workplace, prevention focus, which primarily concentrates on an individual’s fulfillment of responsibilities and obligations, is likely to be one of the key mediators linking an individual graduate’s zero-sum construal of workplace success and his or her initial work role behavior in the context of China’s present competitive job-hunting market and workplace.

First, the individual graduate’s zero-sum construal of workplace success may be an important factor in activating his or her prevention focus inclination. According to regulatory focus theory (Higgins, 1997, 1998), “prevention focus” and “promotion focus” are the two fundamental types of self-regulating mechanisms that depict an individual’s behavioral motives to seek pleasure and avoid pain. Compared to “promotion focus,” “prevention focus” pays more attention to security needs rather than growth needs, rules and responsibilities rather than ambitions, and loss/cost rather than gain. As a result, an individual within a much stronger prevention focus inclination is more sensitive to the occurrence of negative outcomes, is more alert to the fulfillment of personal responsibilities and obligations, and always tries his or her best to avoid mistakes, punishment, and failures (Neubert et al., 2008). It is found that contextual factors and clues are the important triggers of an individual’s self-regulating motives (Crowe and Higgins, 1997). In detail, when the situation tends to encourage an individual to be more concerned with security needs and loss or emphasizes his or her fulfillment of responsibilities and obligations heavily, it is more likely to elicit the individual’s prevention focus inclination. Actually, an individual graduate’s zero-sum construal of workplace success is not a kind of situation, but it is his or her general zero-sum cognition or interpretation of winning in the fierce job-seeking competition or at the workplace, which is essentially the zero-sum construal of the job-seeking situation or workplace situation. Once the individual graduate’s zero-sum construal of workplace success is shaped, it may be more important than specific situations, since it will efficiently guide his or her workplace behavior in a less time- and energy-consuming manner and effectively promote his or her workplace adaptability (Niedenthal et al., 1985; Baldwin, 1992; Fiske, 1992; Weick, 1995). Thus, the individual graduate’s zero-sum construal of workplace success may be one important factor that can activate his or her prevention focus inclination. Furthermore, as zero-sum belief emphasizes the risk and loss more than does positive-sum belief, an individual graduate’s having a stronger zero-sum construal of workplace success often implies that he or she has a higher risk perception and is more sensitive to negative outcomes. This is exactly consistent with prevention focus, which is mainly concerned with prevention goals and punishment-avoidance. Accordingly, the stronger the individual graduate’s zero-sum construal of workplace success is, the higher his or her prevention focus level may be. To sum up, we posit that an individual graduate’s zero-sum construal of workplace success may be more likely to evoke his or her prevention focus inclination.

Second, an individual graduate’s prevention focus evoked by his or her zero-sum construal of workplace success in the context of China’s steady economic development and competitive human resource market may be beneficial to his or her initial work role behavior as a new employee. In general, an individual within a stronger prevention focus inclination is more sensitive to negative information and outcomes, such as mistakes, failures, punishment, cost, and loss (Higgins and Tykocinski, 1992), often pays more attention to the security, rules, duties, responsibilities, and obligations (Kark and Van Dijk, 2007), cares about what one “should” do strongly, and will do his or her best to perform his or her duties, responsibilities, and obligations in order to ensure that his or her actions are in line with expectations (Higgins, 1997, 1998). Initial work role behavior, which refers to the individual graduate’s initial job duties, responsibilities, and tasks and is the crucial factor in workplace adaptability, is just what he or she “should” do in the current working position. Therefore, we predict that an individual graduate’s prevention focus is more likely to promote his or her initial work role behavior, such that the stronger the prevention focus inclination is, the higher the level of initial work role behavior will become. In addition, initial work role behavior is a typical kind of in-role behavior. The extant literature has confirmed that an employee’s prevention focus significantly promotes his or her in-role performance (Neubert et al., 2008), which provides direct evidence for our prediction.

Taking the above two predictions together, we hypothesize:

*Hypothesis 2*: The relationship between an individual graduate’s zero-sum construal of workplace success and initial work role behavior is partially mediated by prevention focus.

### Moderation Role of Pay Level

Higgins (2000) argued that the provision of loss information is more likely to elicit an individual’s prevention focus inclination. As for our study, we believe that an individual graduate’s average pay level to some extent may play a role of information provision and moderate the association between zero-sum construal and initial work role behavior via prevention focus, such that when an individual graduate tends to view workplace success as a zero-sum game, his or her average pay level often represents the opportunity cost of failure in the zero-sum game, and the higher the average pay level is, the larger the opportunity cost of failure in workplace competition will be. This situation is more likely to activate an individual’s prevention actions to avoid potential loss, which in turn strengthens his or her prevention focus level and ultimately promotes initial work role behavior. Conversely, when the individual graduate’s average pay level at the initial working organization is much lower, the opportunity cost of his workplace failure is generally much lower, and the new employee’s prevention focus level will be lower as well, which thereby leads to a lower level of initial work role behavior. To sum up, we propose that an individual graduate’s average pay level may play a moderating role in the relationship between zero-sum construal of workplace success and initial work role behavior via prevention focus.

*Hypothesis 3*: An individual graduate’s average pay level strengthens the positive relationship between zero-sum construal of workplace success and initial work role behavior mediated by prevention focus, such that the positive mediating effect is much stronger when his or her average pay level is relatively high.

**FIGURE 1:**
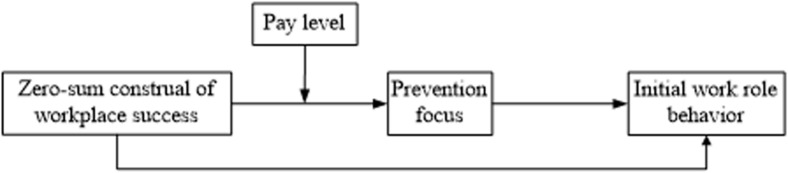
A proposed first-stage moderated mediation model.

Our theoretical model is summarized in Figure 1.

## Materials and Methods

### Sample and Procedure

We chose graduates who had studied at Jiangsu Normal University or Xuzhou University of Technology and had already entered organizations as full-time employees as our research sample. The participants were recruited by sending an email. We sent out a total of 500 emails. After one week, 300 graduates had given responses (response rate = 60%) with the provision of their direct supervisor’s valid email and had agreed to take part in our formal survey, which commenced in December 2018 and ended in May 2019. To avoid the common method variance issue, we asked each participant to self-rate his or her zero-sum construal of workplace success after reporting information about gender, age, organization ownership, and major in December 2018; six months later (i.e., in May 2019), we requested each participant’s direct supervisor to assess his or her initial work role behavior by directly sending an email to the direct supervisor. Meanwhile, each participant self-evaluated his or her prevention focus level and provided the average pay level by month.

We collected 298 questionnaires in all, deleted 40 mismatched ones, and ultimately obtained 258 valid questionnaires. The participants (*N* = 258) were 44.57% male and 55.43% female, with an average age of 23 years; 18.99% came from state-owned organizations; 45.35, 18.99, and 25.97%, respectively, majored in management and economics (major 1), engineering (major 2), and language, psychology, education, and law (major 3).

### Measurement

Following the back-translation procedure (Brislin, 1986), we first translated the English items into Chinese and then back-translated to English items to obtain equivalent meanings. Except for the moderator (i.e., pay level), all the items (in Chinese) measuring the three latent variables (“zero-sum construal of workplace success,” “initial work role behavior,” and “prevention focus”) were evaluated using a five-point Likert rating method that ranges from 1 = strongly disagree to 5 = strongly agree.

Additionally, to guarantee the content validity and reliability of the three latent variables, we administered a pilot survey with an undergraduate sample who had already participated in an internship (*N* = 126). In the pilot survey, each participant self-rated his or her zero-sum construal of workplace success and prevention focus level and his or her direct supervisor in the internship evaluated the level of initial work role behavior. Both used a five-point Likert rating method. The specific items for measuring the three latent variables in the formal survey were retained after the pilot survey.

#### Zero-Sum Construal of Workplace Success

We chose three items that directly focused on to what extent the individual construed “workplace success” in a zero-sum manner from Sirola and Pitesa (2017)’s six-item scale (in which three items focus on “workplace success” and the other three items concentrate on “success” in a broad sense) to measure “zero-sum construal of workplace success.” The three items are “More good jobs for some graduates signifies fewer good jobs for other graduates,” “The more employees an organization employs, the more difficult it is for the extant employees to get a promotion,” and “When some graduates make economic gains, the other graduates lose out economically.” The reliability was 0.98.

#### Initial Work Role Behavior

As a new employee, adaptability is the main tenet of good initial work role behavior. We used three items focusing on individual adaptive behavior (Griffin et al., 2007) to measure initial work role behavior. The three items are “The new employee could adapt well to changes in the workplace,” “The new employee could cope with changes to his or her tasks,” and “The new employee could learn new skills to help him or her adapt to changes in tasks.” The reliability was 0.95.

#### Pay Level

Pay level refers to the individual graduate’s average pay level by month. It was provided by the individual graduate himself or herself. To eliminate the influence of the right-deviated distribution of raw pay data, we ran a logarithmic process before data analysis (He et al., 2016).

#### Prevention Focus

Prevention-focused individuals dedicate attention to security, “oughts,” and loss. We chose three items that separately depicted an individual’s inclination toward security, “oughts,” and loss from Neubert et al. (2008)’s nine-item scale to measure the individual graduate’s prevention focus. These are “Job security is an important factor for me in any job search,” “Fulfilling my job duties is very important to me,” and “I am careful to avoid exposing myself to potential losses at work.” The reliability was 0.94.

#### Control Variables

We controlled for some variables that may be related to prevention focus and initial work role behavior, including gender, age, organization ownership, and major. Moreover, as pay level is found to influence employee attitudes and behaviors (He et al., 2016), we controlled for that as well.

### Statistical Analysis

Other than using the three-step procedure (Baron and Kenny, 1986; Muller et al., 2005), which is mainly based on hierarchical regression analysis within SPSS software, we adopted the Sobel test and Bootstrapping approach in PROCESS (Hayes and Preacher, 2010) to test our first-stage moderated mediation model. Moreover, to ensure the discriminant validity of the three latent variables, we first did a series of confirmatory factor analyses with MPLUS software and Chi-square difference tests between each alternative model and our hypothesized three-factor model.

## Results

Table 1 reports the results of CFA and Chi-square difference tests.

**TABLE 1:** CFA and Chi-square difference test results (*N* = 258).

**Model**	**χ^2^**	**df**	**χ^2^/df**	**RMSEA**	**SRMR**	**CFI**	**TLI**	****Δ**χ (^2^**Δ** df)**
Three-factor	75.57	24	3.15	0.07	0.02	0.98	0.97	−
Two-factor^1^	219.64	26	8.45	0.17	0.04	0.94	0.92	144.07(2)***
Two-factor^2^	931.95	26	35.84	0.36	0.10	0.72	0.61	856.38(2)***
Two-factor^3^	766.04	26	29.46	0.33	0.19	0.77	0.68	690.47(2)***
One-factor	1,095.34	27	40.57	0.39	0.11	0.66	0.55	1,019.77(3)***

***p < 0.001. Three-factor model: zero-sum construal of workplace success; prevention focus; initial work role behavior. Two-factor^1^ model: zero-sum construal of workplace success; prevention focus + initial work role behavior. Two-factor^2^ model: zero-sum construal of workplace success + initial work role behavior; prevention focus. Two-factor^3^ model: zero-sum construal of workplace success + prevention focus; initial work role behavior. One-factor model: zero-sum construal of workplace success + prevention focus + initial work role behavior.

As shown in Table 1, the CFA results indicate that our hypothesized three-factor model has a satisfactory fit to the empirical data: χ^2^(24) = 75.57, χ^2^/df = 3.15, RMSEA = 0.07, SRMR = 0.02, CFI = 0.98, TLI = 0.97, and the hypothesized three-factor model is much better than any of the alternative models, since the Chi-square difference test results are all significant at the 0.001 level.

Table 2 presents the means, standard deviations, correlations, and reliabilities. As shown in Table 2, an individual graduate’s initial work role behavior is positively associated with gender (*r* = 0.14, *p* < 0.05), majoring in management and economics (*r* = 0.18, *p* < 0.01), zero-sum construal of workplace success (*r* = 0.67, *p* < 0.01), and prevention focus (*r* = 0.84, *p* < 0.01), whereas it is negatively related to a major in language, psychology, education, and law (*r* = −0.17, *p* < 0.01) and pay level (*r* = −0.24, *p* < 0.01). Moreover, an individual gradute’s prevention focus is significantly related to gender (*r* = 0.21, *p* < 0.01), a major in management and economics (*r* = 0.19, *p* < 0.01), a major in language, psychology, education, and law (*r* = −0.14, *p* < 0.05), pay level (*r* = −0.27, *p* < 0.01), and zero-sum construal of workplace success (*r* = 0.58, *p* < 0.01).

**TABLE 2:** Means, standard deviations, correlations, and reliabilities (*N* = 258).

	**Mean**	**SD**	**1**	**2**	**3**	**4**	**5**	**6**	**7**	**8**	**9**	**10**
1.C_1_	0.55	0.50	1.00									
2.C_2_	22.77	1.98	0.04	1.00								
3.C_3_	0.19	0.39	–0.06	–0.05	1.00							
4.C_4_	0.45	0.50	0.14**	0.06	–0.04	1.00						
5.C_5_	0.26	0.44	0.09	–0.02	0.03	−0.54**	1.00					
6.C_6_	0.19	0.39	−0.16**	–0.10	0.07	−0.44**	−0.29**	1.00				
7.M_*O*_	8.45	0.42	−0.28**	0.02	0.01	−0.15**	0.11	–0.03	1.00			
8.X	3.08	1.20	0.03	0.04	0.06	–0.02	0.01	0.04	–0.04	(0.98)		
9.M_*E*_	3.29	1.19	0.21**	0.10	–0.06	0.19**	−0.14*	–0.04	−0.27**	0.58**	(0.94)	
10.Y	3.34	1.07	0.14*	0.05	–0.04	0.18**	−0.17**	0.07	−0.24**	0.67**	0.84**	(0.95)

*p < 0.05, **p < 0.01. The reliabilities of X, M_*E*_, and Y are on the diagonal in parentheses. C_1_ means gender, 0 = male, 1 = female; C_2_ means age; C_3_ means organization ownership, 0 = not state-owned, 1 = state-owned; C_4_ means a major in management and economics (major 1), 0 = not in management or economics, 1 = in management or economy; C_5_ means a major in language, psychology, education, and law (major 2), 0 = not in language, psychology, education, or law, 1 = in language, psychology, education, or law; C_6_ means a major in engineering (major 3), 0 = not in engineering, 1 = in engineering. X, M_*O*_, M_*E*_, and Y represent “zero-sum construal of workplace success,” “pay level,” “prevention focus,” and “initial work role behavior,” respectively. The mean and standard deviation of M_*O*_ (pay level) were calculated after a logarithmic process was run on the raw data.

Table 3 presents the results of testing the three hypotheses using hierarchical regression analysis, the Sobel test, and the Bootstrapping approach.

**TABLE 3:** Hierarchical regression analysis, Sobel test, and bootstrapping results (*N* = 258).

	**Prevention focus**			**Initial work role behavior**				
	**Model 1**	**Model 2**	**Model 3**	**Model 4**	**Model 5**	**Model 6**	**Model 7**	**Model 8**
Constant	6.87(1.75)***	6.40(1.36)***	7.03(1.33)***	5.87(1.59)***	5.38(1.11)***	1.68(0.83)*	6.08(1.06)***	2.27(0.82)**
Gender	0.33(0.15)*	0.26(0.12)*	0.27(0.12)*	0.18 (0.14)	0.12 (0.10)	−0.04(0.07)	0.13 (0.09)	−0.02(0.07)
Age	0.05 (0.04)	0.04 (0.03)	0.03 (0.03)	0.03 (0.03)	0.01 (0.02)	−0.01(0.02)	0.00 (0.02)	−0.01(0.02)
Organization ownership	−0.12(0.18)	−0.23(0.14)	−0.18(0.14)	−0.09(0.16)	−0.20(0.12)	−0.06(0.08)	−0.14(0.11)	−0.04(0.08)
Major 1	0.25 (0.26)	0.16 (0.20)	0.14 (0.19)	0.57(0.23)*	0.47(0.16)**	0.38(0.12)**	0.45(0.15)**	0.37(0.11)**
Major 2	−0.17(0.27)	−0.27(0.21)	−0.25(0.20)	0.12 (0.24)	0.01 (0.17)	0.17 (0.12)	0.04 (0.16)	0.17 (0.12)
Major 3	0.06 (0.28)	−0.12(0.22)	−0.30(0.22)	0.59(0.26)*	0.41(0.18)*	0.48(0.13)***	0.21 (0.17)	0.37(0.13)**
Pay level	−0.60(0.18)**	−0.69(0.14)***	−0.73(0.14)***	−0.44(0.16)**	−0.53(0.11)***	−0.13(0.08)	−0.57(0.11)***	−0.18(0.08)*
Zero-sum construal		0.58(0.05)***	0.54(0.05)***		0.60(0.04)***	0.26(0.04)***	0.55(0.04)***	0.26(0.03)***
Zero-sum construal × Pay level			0.29(0.07)***				0.32(0.05)***	0.16(0.04)***
Prevention focus						0.58(0.04)***		0.54(0.04)***
F	5.08***	27.65***	28.15***	4.49***	39.50***	97.06***	43.44***	93.82***
Adjust R^2^	0.10***	0.45***	0.49***	0.09***	0.55***	0.77***	0.60***	0.78***

**The Sobel test and bootstrapping results for the mediation of prevention focus (Hypothesis 2)**								

	**Value**		**SE**	***z***		***p***		

Sobel test	0.34		0.03	9.88		0.00		

**Bootstrapping**	**Effect**		**SE**	**LL 95% CI**		**UL 95% CI**		

Indirect effect	0.34		0.03	0.27		0.41		
Direct effect	0.26		0.03	0.19		0.33		
Total effect	0.60		0.04	0.53		0.68		

**First-stage moderated mediation results for initial work role behavior across different pay levels (Hypothesis 3)**								

**Level**	**Conditional indirect effect**		**SE**	**LL 95% CI**		**UL 95% CI**		

Low (-0.42)	0.18		0.04	0.10		0.26		
High (0.42)	0.47		0.06	0.36		0.58		

*p < 0.05, **p < 0.01, ***p < 0.001. “Prevention focus” is the outcome variable of models 1, 2, and 3, whereas “Initial work role behavior” is the outcome variable of models 4, 5, 6, 7, and 8. Moreover, “major 1” represents a major in management and economics, “major 2” means a major in language, psychology, education, and law, and “major 3” represents a major in engineering. The coefficients for the hierarchical regression analysis (from Model 1 to Model 8) are unstandardized, with standard errors in parentheses. Bootstrapping sample size = 5,000, LL = lower limit, UL = upper limit, CI = confidence interval.

Hypothesis 1 posits that a significant positive relationship exists between an individual graduate’s zero-sum construal of workplace success and his or her initial work role behavior. As reported in Model 5, zero-sum construal of workplace success indeed exerts a significantly positive impact on initial work role behavior (β = 0.60, *p* < 0.001), indicating that the stronger the individual graduate’s zero-sum construal of workplace success is, the higher his or her level of initial work role behavior as a new employee becomes, which supports hypothesis 1.

Hypothesis 2 predicts that an individual graduate’s prevention focus partially mediates the relationship between zero-sum construal of workplace success and initial work role behavior. To test this partial mediation, two approaches were adopted. First, we used hierarchical regression analysis with SPSS software to test whether the three conditions suggested by Baron and Kenny (1986) could hold. The results in Table 3 show that (1) zero-sum construal of workplace success is significantly positive related to initial work role behavior (β = 0.60, *p* < 0.001 in Model 5), which satisfies the first condition that the independent variable (i.e., zero-sum construal of workplace success) should have a significant relationship with the dependent variable (i.e., initial work role behavior); (2) zero-sum construal of workplace success is significantly related to prevention focus (β = 0.58, *p* < 0.001 in Model 2), which meets the second condition that the independent variable (i.e., zero-sum construal of workplace success) should be significantly associated with the mediator (i.e., prevention focus); (3) after controlling for zero-sum construal, the coefficient of prevention focus on initial work role behavior is significant (β = 0.58, *p* < 0.001 in Model 6), and further, the relationship between zero-sum construal and initial work role behavior becomes weaker (from β = 0.60, *p* < 0.001 in Model 5 to β = 0.26, *p* < 0.001 in Model 6), which complies with the third condition that the mediator (i.e., prevention focus) should be significantly related to the dependent variable (i.e., initial work role behavior) when the equation includes the independent variable (i.e., zero-sum construal of workplace success) and the coefficient of the effect of the independent variable on the dependent variable becomes weaker (i.e., a partial mediation) or non-significant (i.e., a complete mediation). Hence, the hierarchical regression analysis results suggest that prevention focus partially mediates the relationship between zero-sum construal of workplace success and initial work role behavior, supporting hypothesis 2. Second, following Hayes and Preacher (2010)’s procedures, we verified hypothesis 2 through the Sobel test and Bootstrapping approach in PROCESS, the results of which are reported after the hierarchical regression analysis results in Table 3. As is shown, the indirect effect of prevention focus is significant (the indirect effect = 0.34, Sobel *z* = 9.88, *p* = 0.00), which is supported by the Bootstrapping results as well. Specifically, the 95% bias-corrected confidence interval for the indirect effect of prevention focus by bootstrapping 5,000 samples is (0.27, 0.41), not including 0. As a result, hypothesis 2 is supported.

**FIGURE 2:**
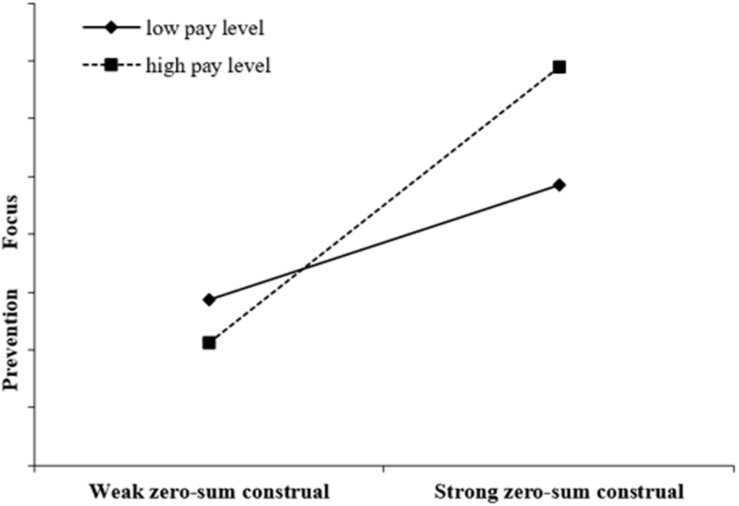
Moderation by pay level.

Hypothesis 3 predicts that the indirect effect of prevention focus in the relationship between an individual graduate’s zero-sum construal of workplace success and his or her initial work role behavior will be strengthened by a high pay level. First, the results of Model 3 in Table 3 demonstrate that the effect of the interaction term (zero-sum construal × pay level) on prevention focus is significant (β = 0.29, *p* < 0.001). Following the procedures recommended by Preacher et al. (2006), we further did a simple slope analysis at 1 SD above and below the mean pay level (see Figure 2). As expected, the simple slope of zero-sum construal of workplace success on prevention focus is less positive (the simple slope = 0.83, *t* = 11.25, *p* < 0.001) when the individual graduate’s average pay level is low (1 SD below the mean), whereas the simple slope of zero-sum construal of workplace success on prevention focus is more positive (the simple slope = 1.99, *t* = 5.88, *p* < 0.001) when the individual graduate’s average pay level is relatively high (1 SD above the mean).

Second, the results of Model 7 in Table 3 show that zero-sum construal is positively related to initial work role behavior (β = 0.55, *p* < 0.001). Third, the results of Model 8 in Table 3 indicate that when controlling for zero-sum construal and the interaction item (zero-sum construal × pay level), the hypothesized mediator (prevention focus) is positively associated with the outcome variable (initial work role behavior; β = 0.54, *p* < 0.001). Therefore, according to the procedures recommended for testing a first-stage moderated mediation (Muller et al., 2005), the above three findings demonstrate that our hypothesized first-stage moderated mediation (i.e., hypothesis 3) is supported. Further, Table 3 also presents the testing results of first-stage moderated mediation using the Bootstrapping approach in PROCESS. As expected, the indirect effect of prevention focus is conditional upon the individual graduate’s average pay level. Specifically, the indirect effect of prevention focus is more positive and significant at a high pay level (the conditional indirect effect = 0.47, 95% CI ranging from 0.36 to 0.58, not including 0) whereas it is less positive and significant at a low pay level (the conditional indirect effect = 0.18, 95% CI ranging from 0.10 to 0.26, not including 0). Hence, hypothesis 3 is established.

## Discussion

The workplace adaptability and sustainable career development of Chinese college and university graduates as new employees have always captured widespread social concern around China. However, few efforts have been made to examine the potential role that an individual graduate’s zero-sum construal of workplace success may play in his or her initial work role behavior and the underlying mechanism. To address this gap, we constructed a first-stage moderated mediation model to explain what effect, why, and under what boundary condition this may occur. Using two-stage survey data from 258 Chinese university graduates who had entered organizations as full-time employees and their direct supervisors, we found that an individual graduate’s zero-sum construal of workplace success not only has a direct and positive effect on initial work role behavior but also exerts an indirect and positive impact via prevention focus. Moreover, a high pay level strengthens the positive mediating effect.

### Theoretical and Practical Implications

Our study has made the following main theoretical contributions. First, it constructs a moderated mediation model to explain individual graduates’ workplace adaptability in the context of China’s present steady economic development, which adds Chinese evidence to the new employee adaptability research and enriches the extant theory. Second, it not only extends the antecedent of the new employee’s workplace adaptability through focusing on the research gap of the potential role of zero-sum construal of workplace success in the new employee’s work role behavior but also enriches research into the new employee’s adaptability mechanism by unraveling the black box from the perspective of prevention focus based on social cognition theory, sense-making theory, and regulatory focus theory. Third, it confirms the “positive” influence of an individual’s zero-sum construal in the context of China’s present steady economic development. Moreover, it examines the moderating role of an individual graduate’s average pay level in the mediating effect of prevention focus in the relationship between zero-sum construal of workplace success and initial work role behavior, which could clarify the boundary condition of the partial mediation.

Regarding the third contribution, we need to make a further explanation. Most of the extant literature tends to emphasize the negative influences of zero-sum construal (Foster, 1965; Esses et al., 1998; Norton and Sommers, 2011; Sirola and Pitesa, 2017), which may blindly cover up its potential positive roles. Undoubtedly, this is not constructive to our comprehensive understanding and correct treatment of an individual’s zero-sum construal. Although we did not empirically explore the antecedent of an individual graduate’s zero-sum construal of workplace success in our research, it was definitely not “cues of economic downturns” [the hypothesized antecedent of zero-sum construal of success in Sirola and Pitesa (2017)’s research], as our research took place in the context of China’s present steady economic development with plenty of job opportunities. The remarkable difference in the backgrounds between Sirola and Pitesa (2017)’s research and our research may be one potential factor leading to the opposite influences of zero-sum construal. In any case, it is demonstrated that zero-sum construal does not always lead to negative effects, as, in some situations, it may have positive effects, which is very worthy of further investigation.

In terms of practical implications, our findings are helpful in solving the unadaptability issue of Chinese college and university graduates as new employees in the workplace and facilitate their sustainable career development. First, based on the direct and indirect positive effects of an individual graduate’s zero-sum construal of workplace success on his or her initial work role behavior, the employment departments of Chinese colleges and universities and the human resource managers of organizations should modify their cognition regarding zero-sum construal and accept the existence of its potential positive roles, as well taking actions to enhance the individual graduate’s zero-sum construal of workplace success and level of prevention focus, such as providing detailed information about the competitive job-hunting market and presenting and emphasizing exact and specific data on loss or cost. Additionally, our study confirms the reinforcing role played by pay level in the mediating effect of prevention focus and meanwhile finds a significantly negative effect on his or her prevention focus and initial work role behavior (see in Table 3). Therefore, managers should weigh the average pay level carefully and reckon whether to change the universally lower average pay level of new employees.

### Limitations and Future Research

First, an individual graduate’s initial work role behavior is so crucial that it is of great significance to explore various kinds of factors that may influence his or her workplace adaptability in both theory and practice. Although we addressed our efforts toward examining the possible effect of “zero-sum construal of workplace success” and “prevention focus” from the perspectives of social cognition and self-regulation motives in the context of China, this is just a drop in a huge ocean. Future research should pay more attention to antecedent studies of new employees’ initial work role behavior and sustainable career development from a much wider variety of perspectives. Further, to extend the external validity, the findings of our research in the context of China need to be examined in other cultural or regional contexts.

Second, we empirically unraveled the partially mediating role of “prevention focus” in the relationship between zero-sum construal of workplace success and initial work role behavior, not taking other potential mediators into consideration. For example, as discussed above, there are two basic self-regulation motives in human beings, including not only “prevention focus” but also “promotion focus.” Our research only explored the potential mediation role of “prevention focus,” as the “zero-sum” nature is exactly consistent with the “loss-attention-bias” of an individual within a stronger prevention focus inclination. However, in addition to “prevention focus,” zero-sum construal of workplace success may influence “promotion focus” as well. Thus, we suggest that future research should consider “promotion focus” or other possible mediators, which will be beneficial for us in comprehensively understanding the underlying mechanism of new employees’ workplace adaptability.

Third, we strongly recommend researchers to further explore the key factors that can bring positive influences of zero-sum construal of success. Our research has confirmed that zero-sum construal of workplace success is positively related to initial work role behavior under China’s present steady economic development, which suggests the existence of positive roles for zero-sum construal. It is very necessary to comprehensively examine the influences of zero-sum construal (whether positive or negative) so as to correctly understand and treat it. Therefore, future research should pay more attention to exploring the key factors that enable zero-sum construal to bring positive effects.

Finally, future research should explore other possible factors that may moderate the relationship between zero-sum construal and prevention focus other than pay level. For example, zero-sum construal itself has a social comparison nature, so individual differences in “social comparison orientation” may influence the triggering effect of zero-sum construal of workplace success on prevention focus (Gibbons and Buunk, 1999).

## Data Availability Statement

All datasets generated for this study are included in the article.

## Ethics Statement

The studies involving human participants were reviewed and approved by the Human Research Ethics Committee (HREC) at the School of Mathematics and Physical Science, Xuzhou University of Technology. Written, informed consent was inferred through the completion of the survey.

## Author Contributions

Both authors contributed equally to constructing the conceptual framework, collecting the empirical data, analyzing the data, and writing the manuscript.

## Conflict of Interest

The authors declare that the research was conducted in the absence of any commercial or financial relationships that could be construed as a potential conflict of interest.
